# Natural essential oils derived from herbal medicines: A promising therapy strategy for treating cognitive impairment

**DOI:** 10.3389/fnagi.2023.1104269

**Published:** 2023-03-16

**Authors:** Ai Shi, Yu Long, Yin Ma, Shuang Yu, Dan Li, Jie Deng, Jing Wen, Xiaoqiu Li, Yuanyuan Wu, Xiaofang He, Yue Hu, Nan Li, Yuan Hu

**Affiliations:** ^1^State Key Laboratory of Southwestern Chinese Medicine Resources, Chengdu University of Traditional Chinese Medicine, Chengdu, China; ^2^College of Pharmacy, Chengdu University of Traditional Chinese Medicine, Chengdu, China

**Keywords:** cognitive impairment, Alzheimer’s disease, natural essential oils, mechanism, advantages

## Abstract

Cognitive impairment (CI), mainly Alzheimer’s disease (AD), continues to increase in prevalence and is emerging as one of the major health problems in society. However, until now, there are no first-line therapeutic agents for the allopathic treatment or reversal of the disease course. Therefore, the development of therapeutic modalities or drugs that are effective, easy to use, and suitable for long-term administration is important for the treatment of CI such as AD. Essential oils (EOs) extracted from natural herbs have a wide range of pharmacological components, low toxicity, and wide sources, In this review, we list the history of using volatile oils against cognitive disorders in several countries, summarize EOs and monomeric components with cognitive improvement effects, and find that they mainly act by attenuating the neurotoxicity of amyloid beta, anti-oxidative stress, modulating the central cholinergic system, and improving microglia-mediated neuroinflammation. And combined with aromatherapy, the unique advantages and potential of natural EOs in the treatment of AD and other disorders were discussed. This review hopes to provide scientific basis and new ideas for the development and application of natural medicine EOs in the treatment of CI.

## 1. Introduction

Population aging has become a serious problem in many parts of the world, and with the advent of population aging, CI, which is closely related to age, has shown a significant increase in recent years. Cognition is an indispensable ability in everyday life that enables people to live easily, solve problems and situations, and continue to learn and correctly process information from the environment for subsequent retrieval and use (Gutiérrez Rodríguez and Guzmán Gutiérrez, 2017). CI is impairment of one or more aspects of cognitive processes, including reduced efficiency or impaired functioning of processes in memory, computation, orientation, structural ability, executive ability, language comprehension and expression, and application, and can range from mild CI to dementia (China expert consensus group on prevention and treatment of cognitive dysfunction, 2006). Neurocognitive disorders, especially major neurocognitive disorders (Disorders and Specialized Committee on Cognitive Disorders, N.B., Chinese Medical Association, 2018), have serious consequences for individuals and families, health care systems, and the economy. From 1990 to 2016, the number of people suffering from dementia has more than doubled worldwide (Hugo and Ganguli, 2014), and in 2019, the number of people with dementia worldwide is estimated to be 57.4 million, which is expected to increase to 83.2 million cases by 2030, and this number is expected to reach 152.8 million in 2050 (GBD 2019 Dementia Forecasting Collaborators, 2022). The World Health Organization released the Global Status Report on Addressing Dementia in Public Health stating that the global cost of dementia is approximately $1.3 trillion in 2019 and is expected to increase to $1.7 trillion by 2030. The prevalence of dementia increases exponentially with age, doubling every 5 years after age 65, with a prevalence of 5 to 10% in high-income countries for people aged 65 and older, and is typically higher in women than in men, in large part because women live longer than men (Jia et al., 2020). At the same time, as people’s lifestyles change and living standards continue to improve, common risk factors such as hypertension, diabetes, atrial fibrillation, stroke, and smoking are increasing, and these risk factors lead to progressive damage to large, medium, and small cerebrovascular arteries, which subsequently cause neurodegenerative pathologies and cognitive dysfunction. As a result, the incidence of CI is expected to continue to increase continuously, it is posing an increasingly serious threat to human health, and is emerging as one of the major health problems of society. However, as of today, only 13 of the 193 member countries of the WTO have national dementia control, and there are no first-line therapeutic drugs for allopathic treatment or reversal of the disease course, which can only alleviate CI in early stage patients and provide moderate symptom improvement, but cannot stop the progression of the disease. Thus, the development of therapeutic modalities and drugs that are effective, easy to use, and suitable for long-term administration is of great importance for the treatment of neurological disorders such as CI.

Volatile oils of natural drugs come from nature and are also known as EOs, which are volatile oily liquids, most of which have aromatic odors and are inexpensive and easily available. In basic and clinical studies, volatile aromatic natural medicines or EOs have been found to relieve tension and anxiety, improve depression, improve cognition, and enhance sleep quality (Guo et al., 2020; Cheong et al., 2021; Dos Reis Lucena et al., 2021; Wang et al., 2021a). AD is a major form of dementia characterized pathologically by the abnormal deposition of β-amyloid (Aβ) outside brain cells leading to senile plaques (SPs) and the hyperphosphorylation of Tau proteins within brain cells leading to neuronal fibrillary tangles (NFTs; Durairajan et al., 2022). Aβ self-aggregation and abnormal aggregation of Tau proteins form SPs and NFTs, which in turn disrupt the structure and function of neuronal cells, impairing the antioxidant balance of cells, causing oxidative stress, inflammatory immune response, and mitochondrial energy disorders, further promoting Aβ deposition and exacerbating cognitive dysfunction, while pro-inflammatory factors released by glial cells exacerbate the inflammatory response, leading to severe loss, degeneration and functional deficits of cholinergic neuronal sites, damaging acetylcholinergic nerves, and leading to impaired learning and memory and cognitive impairment. The therapeutic effects of volatile oils on CI such as AD have been reported in several papers. This paper reviews and summarizes the plant volatile oils and monomeric compounds with cognitive improvement effects, as shown in Tables 1 and 2. The results indicate that plant volatile oils mainly exert their effects on controlling AD and improving cognition through their compounds such as monoterpenes, sesquiterpenes and phenylpropanoids by inhibiting the deposition of Aβ, hyperphosphorylation of Tau, and regulating the central cholinergic system, thereby exerting a response state against inflammation and oxidative stress. Thus the natural drug volatile oil may be a potential novel drug for the treatment of cognitive impairment, and aromatic substances sniffing may be a new way to prevent or delay cognitive impairment at an early stage, which has a promising future in the field of cognitive dysfunction treatment.

**Table 1:** Anti-CI effects and mechanisms of the EOs isolated from natural plants.

Source plant	Active ingredients	Dose/concentration	*In-Vivo* model	*In-Vitro* model	Effects and mechanisms	Ref.
*Ligusticum chuanxiong* hort	Senkyunolide A; Ligustilide	i.g.; 30,15,7.5 mg/kg, for14 days;	Mice; VCI induced by LPS	BV-2 microglial cells	Significantly reducing the levels of MAO and AchE in the brain of VCI mice; inhibiting the proliferation of BV-2 cells and reducing the increase of inflammatory factors TNF-α, NO levels.	Zhou et al. (2019)
*Coriandrum sativum* var.	Linalool (69.358%); γ-terpinene (7.729%); α-pinene (6.509%)	i.a.; 1 and 3%	Rats; the Aβ1-42-induced AD		Decreasing SOD and LDH specific activities, increasing GPX specific activity and attenuating the increased MDA level, Provides neuroprotection by alleviating oxidative stress induced by Aβ1-42 injection; reducing amyloid deposits in the hippocampus.	Cioanca et al. (2013), Cioanca et al. (2014)
*Angelica sinensis (Olive.)* Diels		i.g.; 75 mg/kg/day; for 4 weeks	Rats; VCI		Promoting the expression of neuron-protective Bcl-2 protein, reducing the expression of apoptotic Bax protein in brain tissue, thereby inhibiting neuronal apoptosis and accelerating the recovery of neurological function.	Cui (2015)
*Cinnamomum cassia* Presl	phenylallyl compounds	i.g.; 15 days	APP transgenic AD mouse		Inhibiting the increase of COX activity and increasing the release of prostaglandin E2, thus improving the memory function of APP transgenic AD mice.	Ran et al. (2017)
*Thymus vulgaris* L.	Thymol (42.10%); cymene (19.20%); β-caryophyllene (6.40%); Carvacrol (2.70%); α-Pinene (1.52%)	Immersion; 25,150,300 μl/L; for 13 days	Sco-induced zebrafish (Danio rerio) model of memory impairments		Anti-oxidative stress and inhibition of AChE activity.	Capatina et al. (2020)
*Chimonanthus nitens* Oliv.leaves		i.g.; 19.60,4.90 mg/kg, for 28 days	Rats; Replication of VD model by 2VO		Increasing SOD activity and decreasing MDA content, thereby reducing the damage to the organism from oxidative stress, as well as the extent of neuronal cell damage in the CA1 region of the hippocampus.	Wang et al. (2021b)
*Magnolia denudate* Desr.		i.a.; for 2 weeks	Mice; VPA induced ASD model		Improving the learning memory behavior and increasing the expression of 5-HT and DA proteins in ASD model rats, the mechanism may be related to the excitability of olfactory transmission pathways and functional brain regions such as hippocampus, amygdala and hypothalamus, as well as the plasticity of neural circuitry.	He (2016)
Citrus × limon (Linnaeus) Osbeck		i.a.; 1 ml/cage,1 h; for 30 days	Mice; (APP/PS1) AD		decreasing neuronal loss, improving learning and memory ability in APP/PS1 mice after neurodegeneration, suppressing accumulation of amyloid protein, downregulating AChE activity in the hippocampus and Enhancing synaptic plasticity by increasing BDNF, PSD95 and synaptophysin to improve memory levels.	Liu et al. (2020)
*Schisandra chinensis* Baill.	β-Terpinene (19.50%); 1R-α-pinene (3.60%); Benzene,1-methyl-2-(1-methylethyl) (4.63%); Terpinen-4-ol (4.92%); Benzene,2-methoxy-4-methyl-1-(1-methylethyl) (4.57%); Nerolidol (6.71%); (−)-g-Cadinene (4.32%)	i.g.; 0.2,0.067 g/kg once a day	Rats; AD induced by ICV Aβ (1–42)		Improving the activities of SOD, MDA, GSH-Px.	Yang et al. (2018)
*Schisandra chinensis* (Turcz.) Baill.		i.g.; 120 mg/kg; for 28 days; 6.25,12.5,25,50 μg/ml for 2 h	Mice; AD induced by ICV Aβ1(−42) AD induced by i.p. LPS	BV-2 microglial cells	Inhibiting the activation of NF-κB/MAPK pathway activation, reducing the phosphorylation of p-38 and attenuating the release of pro-inflammatory cytokines IL-1β, 1 l-6, and TNF-α, as well as improving microglia activation.	Xu et al. (2019)
*Tetraclinis articulata*	camphor (14.52%); α-pinene (22.68%); L-bornyl acetate (16.87%); borneol (5.2%); limonene (7.34%)	i.a.; 200 μl,1,3%; for 21 days	Rats; AD induced by ICV Aβ1-42 to right-unilaterally		Increasing the activity levels of SOD, CAT and GPX in hippocampal tissue, significantly ameliorating the Aβ1-42-induced decrease in GSH levels and increasing and MDA, thereby reducing oxidative stress in the rat hippocampus to ameliorate the memory deficit induced by Aβ1-42 treatment.	Sadiki et al. (2019)
*Pinus halepensis*	beta-caryophyllene (29.45); pinene (11.14%); myrcene (7.85%); terpinolene (3.90%); 2-phenylethylisovalerate (10.38%); alpha-humulene (6.49%)	i.a.; 200 μl,1,3%; for 21 days	Rats; AD induced by ICV Aβ1-42		Inhibiting of AChE activity and reducing oxidative damage in rat hippocampus.	Postu et al. (2019)
*Chamaecyparis obtusa* Sieb. & Zucc.	α-terpinyl acetate (16.82%); β-phellandrene (13.11%); β-myrcene (5.68%); limonene (6.49%); bornyl acetate (7.48%); γ-terpinene (4.28%); α-terpineol (4.33%); elemol (6.22%); thujopsene (4.50%); β-eudesmol (4.13%); beyerene (3.35%)	i.a.;1 ml/cage,2 h;for 30 days	Rats; AD induced by ICV Aβ1-40		Inhibiting neuronal apoptosis and AChE activity.	Bae et al. (2012)
*Rosa rugosa* Thunb.	6-Octen-1-ol,3,7-dimethyl-,(R)-(+)-Citronellol (54.02%); 2,6-Octadien-1-ol,3,7-dimethyl-,(E)-trans-Geraniol (15.01%)	2 μg/ml,10 μg/ml,20 μg/ml		C. elegans	Suppressing Aβ deposits and reducing the Aβ oligomers to alleviate the toxicity induced by Aβ overexpression, activating the expression of GST-4 gene, which may act through SKN-1 signaling pathway.	Zhu et al. (2017)
*Zataria multiflora* Boiss.		i.p.; 50,100,200 μl/kg;	Rats: AD induced by ICV Aβ25–35		Antioxidant, anti-inflammatory and anticholinesterase activity.	Majlessi et al. (2012)
*Lavandula angustifolia* Mill.	Linalool (33.1%); linalyl acetate (10.4%); 1,8-cineole (8.0%); borneol (4.5%)	i.p.; 50, 100 mg/kg/d; for 10 days 5, 10, 25, and 50 μg/ml; for 24 h	Mice; sco-induced cognitive deficits	PC12 cells exposed to Aβ1-42	Protecting cells free from Aβ1-42 oligomer-induced molecular damage, inhibiting activation of the pro-apoptotic enzyme caspase-3 and the increase of intracellular ROS; inhibiting the AChE activity; exerting anti-oxidative stress effects.	Xu et al. (2016), Caputo et al. (2021)
*Rosmarinus officinalis*	α-pinene (11.1%); camphene (4.8%); β-pinene (6.4%); 1,8-cineole (46.0%); camphor (10.8%); borneol (2.8%); (E)-caryophyllene (3.0%)	i.a.; 20 μl or 40 μl; for 90 min	Mice; AD induced by scopolamine		Producing a significant improvement in the rate of spontaneous alternation behavior, activating of CNS to improve cognitive function.	Satou et al. (2018)
*Thymus vulgaris* L.	Thymol (42.10%); p-cymene (19.20%); β-caryophyllene (6.40%); Carvacrol (2.7%).	Immersion;25,150, 300 μl/L;for 13 days	Sco-induced zebrafish (Danio rerio) model of memory impairments		Ameliorating Sco-induced increasing of AChE activity, amnesia, anxiety, and reducing the brain antioxidant capacity.	Capatina et al. (2020)
*Mentha piperita* Linn.	Menthol (45.56%); menthone (20.9%); menthol acetate (6.64%); 1,8-cineole (4.77%); new menthol (3.27%); iso-menthone (3.08%); menthofuran (2.05%); β-caryophyllene (1.79%); limonene (1.48%); pulegone (1.31%); germacrene D (1.17%)	2 times/day, 1 h/time, for 21 days	APP/PS1 transgenic mice		Reducing Aβ deposits in the brain, protecting neuronal cells and restoring them to their normal state, and reducing peroxidative damage to brain tissue, it may improve cognitive function in AD by regulating arginine and proline metabolism, inositol phosphate metabolism, and cysteine and methionine metabolism.	Lv et al. (2022)
*Alpinia Oxyphylla* Miq.	1,2,4,5-tetramethylbenzene (42.96%); myrtenal (4.66%); linalool (4.34%); (−)-4-terpineol (2.96%); g-terpinene (2.21%); (+)-nootkatone (1.48%); β-pinene (1.32%); (+)-(4R)-limonene (1.25%); (1S)-(+)-3-carene (1.02%)	i.g.; 0.5,1 ml/kg; for 27 days	Mice; sco-induced learning and memory impairment		Regulating the activity of ACh synthase and catabolic enzymes, improving the antioxidant capacity of the body, up-regulating the expression of BDNF, ERK, CREB, Bcl-2 and other genes and proteins p-ERK1/2 and p-AKT473, and down-regulating the expression of Bax and caspase-3 proteins, the mechanism of which may be related to the regulation of hippocampal neuronal apoptosis.	Ma et al. (2018)
*Punica granatum* L.		1.6%Nano-PSO	5XFAD mice		Reducing accumulation of Aβ and p25, a calpain product, and increasing expression of COX IV-1, a key mitochondrial enzyme.	Binyamin et al. (2019)
*Acorus tatarinowii* Schott	β-asarone (54.62%); α-asarone (32.34%)	i.g.; 15 mg/kg, 30 mg/kg,60 mg/kg; for 30 days; 1 mg/ml	APP/PS1 double transgenic rats,	C.elegans	Inhibiting the conversion of Aβ25-35 from α-helix to β-fold and affecting its secondary structure, thus preventing Aβ aggregation and fibril formation; increasing ChAT levels, decreasing GFAP expression and protecting neurons in hippocampal tissue; reducing the deposition of misfolded Aβ and polyQ proteins and improving serotonin sensitivity and olfactory learning skill in worms, its maintenance of protein homeostasis depends on an autophagic pathway regulated in part by the hsf-1 and sir-2.1 genes.	Ma et al. (2007) Deng et al. (2019), Chen et al. (2020)
SuHeXiang Wan Essential Oil	Benzyl Benzoate (29.87%); isobutyl cinnamate (3.05%); 17-oxygen lupinine (2.80%); Benzylcinnamic acid (2.53%); caryophyllene (2.42%); acetophenonepropyl ester (1.83%); Benzyl acetate (1.71%)	i.a.;for 14 days; 1,10,100 μg/ml;for 24 h	Mice;cognitive deficits induced by Aβ1-42	SH-SY5Y cell induced by Aβ1-42	Inhibiting Aβ-induced apoptosis and ROS production by upregulating HO-1 and Nrf2 expression; inhibiting Aβ-induced Tau phosphorylation by inhibiting JNK and p38 activation in the brain; promoting Bcl-2 expression and inhibiting Bax expression thereby inhibiting apoptosis.	Jeon et al. (2011)
*Listea cubeba* (Lour.) Persoon	d-limonene (14.15%); β-myrcene (3.04%); methylhepteneone (2.15%); geranial (31.74%); neral (30.94%)	p.o.	Mice;AD induced by CV Aβ1-40		Inhibiting levels of oxidative stress (including MDA and phosphorylated tau protein) in the brain and preventing brain atrophy.	Lee et al. (2021)
Essential Oil Mix	Limonene (91.11%); γ-terpinene (2.02%); β-myrcene (1.92%); β-pinene (1.76%); α-pinene (1.01%); sabinene (0.67%); linalool (0.55%); cymene (0.53%); valencene (0.43%)	i.a.; 1,3%; for 21 days	Rats; Sco-induced Amnesia		Restoring the activity of the cholinergic system and the antioxidant status of the brain.	Boiangiu et al. (2020)

2VO, Permanent ligation of bilateral common carotid arteries; 5-HT, 5-hydroxytryptamine; Aβ, beta amyloid peptide; AKT, protein kinase B; ASD, autism spectrum disorder; BDNF, brain-derived neurotrophic factor; Bax, Bcl-associated X; Bcl-2, B-cell lymphoma-2; COX, Cyclooxygenase; CAT, catalase; CREB, cAMP-response element binding protein; ERK, extracellular regulated protein kinases; GPX, Glutathione Peroxidase; GFAP, glial fibrillary acidic protein; HCl, Hydrochloride; HO-1, heme oxygenase-1; i.a., inhalation; i.g., intragastric; i.p., intraperitoneal; JNK, c-Jun Loxl2, MAO, Monoamine oxidase; MDA, malondialdehyde; NMDA, N-methyl-D-aspartate; LDH, lactate dehydrogenase; LPS, lipopolysaccharide; NGF, Nerve growth factor; Nrf2, erythroid-derived 2-related factor 2; NO, Nitric oxide; PSD95, post-synaptic density protein 95; ROS, reactive oxygen species; Sco, Scopolamine; TNF-α, tumor necrosis factor-α.

**Table 2:** Anti-IOF effects and mechanisms of the monomers isolated from natural plants.

Classification	Compound name	Source plants	Model	Dose and duration	Mechanism	Ref.
Sesquiterpenes	Bergapten (5-methoxypsoralen)	*Citrus × limon* (Linnaeus) Osbeck; *Citrus medica* L.var.sarcodactylis Swingle	Mice; Sco-induced Memory Impairment	i.p.; 12.5,25,50,100 mg/kg; for 6 days	Inhibiting AChE activity in hippocampus and prefrontal cortex with some antioxidant ability.	Kowalczyk et al. (2020)
	α-Asarone	*Acorus tatarinowii schott.*	Mice; ethanol-induced learning and memory impairment	i.p.; 7.5,15,30 mg/kg; for 6 days	Regulating the expression of Glu and GABA and related proteins in the hippocampus.	Li et al. (2019)
	β-asarone	*Acorus tatarinowii schott.*	APP/PS1 transgenic mice; Rats; AD induced by β-amyloid; PC12 cells	i.g.; 10,20,40 mg/kg; for 30 days; i.g.; 12.5,25,50 mg/kg;for 50 days	Providing protection against oxidative stress and neuronal damage induced by β-amyloid;decreasing AChE, Aβ1-40, and Aβ1-42 levels, increasing p-mTOR and p62 expression, decreasing p-Akt, Beclin-1, and LC3B expression, decreasing the number of autophagosomes and reducing APP mRNA and Beclin-1 mRNA, It inhibits autophagy by regulating the PI3K/Akt/mTOR/Beclin-1 pathway.	Deng et al. (2016), Deng et al. (2020), Li et al. (2021)
	β-Caryophyllene	*Piper nigrum*	Mice;Sco-induced Amnesia	p.o.; 50,100 mg/kg; for 14 days	Reducing proBDNF/mBDNF ratio and increasing TrkB expression, reducing Scop-induced upregulation of p-JNK and p-p38 MAPK proteins, Bax/Bcl-2 ratio and caspase activation in the brain, and downregulating Cox-2, TNF-α and NOS-2 to exert anti-inflammatory effects.	Sudeep et al. (2021)
	Oxyphylla A	*Alpinia oxyphylla Miq.*	N2a/APP cells; SAMP8 mice	p.o.; 10,20 mg/kg; for 6 weeks 200 mM;24 h	Reducing the expression levels of APP and Aβ proteins and exerting antioxidant effects through the Akt-GSK3β and Nrf2-Keap1-HO-1 pathways.	Bian et al. (2021)
	α-Cyperone	*Cyperus rotundus L.*	LPS-induced BV2 cells	15,30,60 μM; 24 h	Up-regulating Nrf2, HO-1, p-Akt, down-regulating p-NF-κB, p65, TNF-α, IL-6, IL-1β. inhibiting the production of inflammatory cytokines in BV-2 cells by activating Akt/Nrf2/HO-1 and inhibiting NF-κB pathway, thus exerting neuroprotective effects.	Huang et al. (2018)
Monoterpenes	Cuminaldehyde	*Cuminum cyminum L.*	9 months-old mice	i.g.; 25 mg/kg;for 30 days	Up-regulating the gene expression of BDNF, Icam, and APOE, and down-regulating the expression of IL-6.	Omari et al. (2021)
	Terpinolen	*Satureja hortensis, Pseudotsuga* Menziesii	Rats; AD induced by ICV Aβ1-42	p.o.;100 mg/kg;for 2 weeks	Reducing amyloid plaque counts and ameliorating biochemical factors (higher levels of SOD and MDA)	Bahareh et al. (2020)
	Limonene	*Citrus limon* (L.) Burm. f.	PC12 cells;Cortical neurons;SH-SY5Y cells	5,10,25 μg/ml;24 h	Counteracting the increase of ROS production triggered by Aβ1-42 oligomers, thus preventing the upregulation of KV3.4 activity. In turn, preventing cell death of primary cortical neurons exerting neuroprotective effect against Aβ1-42-induced toxicity.	Piccialli et al. (2021)
	1,8-cineole	*Eucalyptus globulus(E. globulus)*	Differentiated PC12 cells treated with Aβ25-35	2.5, 5 and 10 μM, 24 h	Restoring cell viability, reducing mitochondrial membrane potential, ROS and NO levels, and decreasing the expression of TNF-α, IL-1β, IL-6, iNOS, COX-2 and NF-κB.	Khan et al. (2014)
	Linalool	*Lavandula angustifolia*, *Melissa officinalis*, *Rosmarinus officinalis*, *Cymbopogon citratus*	Mice;3xTg-AD	p.o.;25 mg/kg;48 h for 3 months	Counteracting Aβ1-42 oligomer-induced decrease of mitochondrial dehydrogenase activity, decreasing intracellular ROS production and activation of caspase-3; delaying cerebral amyloidosis, including amyloid deposits and β-amyloid peptide abundance, decreasing PHFs in the CA1 area, suggesting a reduction in NFTs, reducing the levels of IL-1β, iNOS, COX-2, p38 MAPK.	Sabogal-Guáqueta et al. (2016), Yuan et al. (2021)
Diterpenoids	Ginkgolide B	*Ginkgo biloba L.*	OPC after white matter lesion	i.p.;5,10,20 mg/kg, for 4 weeks	Promoting the differentiation of OPC into oligodendrocytes, reducing the apoptosis of oligodendrocytes and the loss of myelin, and enhancing the expression of p-Akt and CREB, thus improving the learning and memory ability of rats with cerebral white matter lesions.	Huang et al. (2021)
Others	Ligustilide	*Ligusticum chuanxiong* hort, *Angelica sinensis* (Olive.) Diels	Rats; VD induced by 2VO	p.o.;20,40 mg/kg/day;for 4 weeks	Inhibiting the increase of Bax and cleaved caspase 3, promoting the expression of Bcl-2 protein, and enhancing the expression of P-AMPK and Sirtuin1 (SIRT1) in VaD rats, the mechanism may be related to the activation of AMPK/SIRT1 pathway; regulating mitochondrial dysfunction and mitochondrial related inflammation, induction of α-Secretase processing of both APP and Klotho, regulating endoplasmic reticulum stress and autophagy;attenuating apoptosis, inhibiting the increase in intracellular ROS accumulation, and reversing the inhibition of PI3-K/Akt pathway to counteract Aβ-induced neurotoxicity	Zhu et al. (2020), Peng et al. (2022a, 2022b)
	6-Gingerol	Z*ingiber officinale* Rosc.	Mice; Sco-induced Amnesia	p.o.;10,25 mg/kg/day;for 3 days	Elevating BDNF protein expression by activating the Akt/CREB signaling pathway.	Kim et al. (2018)
	Thymoquinone	*Nigella damascena* L.	Rats;D-gal and aluminum chloride induced neurotoxicity	i.g.;20 mg/kg /day;for 14 days	Increasing SOD, TAC, decreasing MDA, NO levels and AChE activities as well as TNF-α immunoreactivity and increasing BDNF and Bcl-2 levels as well as ACh immunoreactivity.	Abulfadl et al. (2018)

Akt, protein kinase B; APOE, apolipoprotein E; COX-2, Cyclooxygenase-2; GABA, Gamma amino butyric acid; Glu, glutamate; Icam, intercellular adhesion molecule; iNOS, inducible nitric oxide synthase; Keap1, Keleh-like ECH-associated protein; mTOR, mammalian target of rapamycin, mTOR; MAPK, mitogen-activated protein kinase; OPC, oligodendrocyte precursor cell; PI3K, phosphatidylinositol-3-kinase; PHFs, pair helical filaments; p38 MAPK, p38 mitogen-activated protein kinase.

In this review, we summarize the available drugs for the treatment of cognitive disorders or anti-dementia, review the history of the use of plant volatile oils to combat cognitive disorders in some countries, and then summarize the material basis of natural plant volatile oils to improve cognition, and explain the mechanism of their cognitive improvement effect, hoping to provide a theoretical basis for future researchers to explore more suitable routes of administration and forms of formulation for the development of potential anti-cognitive drugs.

## 2. Available drugs for cognitive impairment and adverse effects

At present, the treatment of cognitive disorders such as dementia is mainly symptomatic and there is no exact and effective treatment. According to multinational guidelines (O’Brien et al., 2017; Disorders and Specialized Committee on Cognitive Disorders, N.B., Chinese Medical Association, 2018; Kandiah et al., 2019; Ismail et al., 2020) the listed anti-dementia drugs are mainly classified as cholinesterase inhibitor (ChEI), such as donepezil, carboplatin, galantamine, Huperzine A; N-methyl-D-aspartate receptor antagonists, such as meperidine hydrochloride; antioxidants, such as Ginkgo biloba extract; pro-intellectual drugs, such as olacetam, piracetam, aniracetam; ergot alkaloids, such as dihydroergotoxine mesylate, nicergoline and other drugs. The main risk factors for CI such as AD, VCI, and diabetes-related cognitive impairment are summarized in Table 3, A summary of current clinical medications for the treatment of cognitive disorders was made and is shown in Table 4.

**Table 3:** Risk factors related to cognitive impairment (Li et al., 2014; Gottesman et al., 2017; Walker et al., 2017; Zheng and Chen, 2018; Gannon et al., 2019; Silva et al., 2019; Duan and Wen, 2020; Lyu et al., 2020).

Cognitive impairment classification	Associated risk factors
VCI	Hyperhomocysteinemia, carotid atherosclerosis, hypertension, diabetes mellitus, hyperlipidemia, hyperuricemia, coronary artery disease, cerebral white matter lesions, stroke, cerebral atrophy, atrial fibrillation, arrhythmia, advanced age, history of smoking
AD	Abnormal blood pressure, dyslipidemia, diabetes, sleep disorders, abnormal metal ion metabolism, depression, poor lifestyle, hyperlipidemia, hypertension, cerebrovascular disease, obesity, metabolic syndrome, traumatic brain injury, depression, advanced age, smoking, low educational level, lack of exercise
Cognitive dysfunction in diabetes	Glucose metabolism disorders, insulin resistance, abnormal adipocyte secretion factors, disruption of calcium homeostasis, sleep disorders, lack of exercise, stroke

**Table 4:** Available drugs for CI and adverse effects.

	Classification	Representative drugs	Adverse reactions	Indications
Drugs acting on the cholinergic system	Cholinesterase inhibitors	Tacrine, Donepezil, Rivastigmine, Galanthamine, Huperzine A.	Common adverse reactions include nausea, vomiting, diarrhea, fatigue, lethargy, muscle cramps, lack of appetite, abdominal pain, weight loss, etc. Occasionally, dizziness, headache, mental disturbances (hallucinations, agitation, aggressive behavior), depression, excessive dreaming, drowsiness, loss of vision, chest pain, gastrointestinal disorders, rash, urinary frequency, urinary tract infection, etc.	Mild and moderate AD
	
Drugs acting on the non-cholinergic system	NMDAR antagonists	Memantine	Common adverse effects such as confusion, constipation, dizziness, headache, confusion, fatigue, etc.	Moderate to severe AD
	Cerebral vasodilator drugs (Calcium antagonists)	Nimodipine, Nicardipine, Flunarizine HCl, Flunarizine, Nitrendipine	Adverse reactions such as flushing, dizziness, headache, itching of the skin, numbness of the mouth and lips, rash, drowsiness, lethargy, palpitations, dry mouth, nausea, ankle edema, mild reflex heart rate acceleration, weight gain, lack of appetite, etc.	Adjunctive therapy VD
	brain metabolic activator	Oxiracetam, Piracetam, Aniracetam	Dry mouth, loss of appetite, vomiting, insomnia, excitement or rash are rarely seen; insomnia, dizziness, vomiting and overexcitement may occur in high doses and disappear on their own after stopping the drug.	Mild to moderate VD, AD, and memory and intellectual impairment caused by traumatic brain injury
	Ergot alkaloids	Hydergine, Nicergoline	Minor adverse reactions such as nausea, vomiting, lack of appetite, stomach pain, diarrhea, facial flushing, hot flashes, dizziness, insomnia, hypotension, tinnitus, and lethargy may be seen occasionally	Symptoms of functional and intellectual decompensation following acute and chronic cerebrovascular disease; mild to moderate VD, AD
	Antioxidants, anti-inflammatory drugs	Vitamin C, Vitamin E, Monoamine Oxidase Inhibitors, Idebenone, Melatonin, Edaravone, Aspirin, Indomethacin, Tenidap	Allergic reactions, rash, nausea, loss of appetite, diarrhea, excitement, insomnia, dizziness, occasionally leukopenia, hepatic impairment. Edaravone can cause abnormal liver function, rash, severe acute renal failure, DIC, etc.	Delayed onset of Alzheimer’s disease; mild to moderate VD, AD
	Neurotrophic drugs	NGF, estrogen, Ganglioside, Pyrithioxin dihydrochloride, Ginkgo leaf preparation	In a few cases, headache, rash, drug fever, diarrhea, loss of appetite and nausea occurred after taking the drug, which can be recovered after stopping the drug.	Aids in improving Alzheimer’s symptoms
	others	Effendil		Dementia caused by cerebrovascular disease (cerebral atherosclerosis, etc.)

## 3. History and modern expansion of essential oil for cognitive disorders

The use of aromatic herbs and plants to treat various disorders of the mind and body has been documented long ago, and certain medicinal herbs are the source of the aromatic oils refined today. Volatile oils have been used in many countries and nations to exert a waking effect and improve cognition, as shown in Table 5. In classical Chinese medical texts, the use of intranasal administration of aromatic substances for the treatment of confusion is well documented, for example, in the “Li Yue Pian Wen,” it is written that people who suffer from unconsciousness during a stroke can use Croton tiglium oil and soapberry powder to smoke their noses to wake them up, while in India, Iran, and other countries, there are also applications of natural medicines such as volatile oils to improve cognition, and these long-term uses have provided a strong basis for modern research on volatile oils For example, modern pharmacological studies have shown that Kaixin Powder can improve psycho-behavioral symptoms of AD by adjusting transmitter homeostasis, inhibiting inflammation, protecting mitochondria, and reducing neuronal damage; Yuanzhi Powder can improve psycho-behavioral symptoms of AD by inhibiting oxidative damage, reducing Tau protein phosphorylation, and It can exert puzzling effects by inhibiting oxidative damage, reducing Tau protein phosphorylation, and regulating cholinergic effects. Studies on the action of these formulas and the volatile oils in them, and modern mechanisms we have also summarized in Table 5.

**Table 5:** Historical and modern extensions of volatile oils in the treatment of cognitive disorders.

Classic, country, or nation	Drug	Main ingredients	Effects and modern mechanisms	Ref.
*Valuable Prescriptions for Emergency*	Kaixin Powder	α-Asarone, β-asarone	Mental-tranquilization, Invigorating The Brain And Ichihing Fruit Reduce the levels of inflammatory factors TNF-α, IL-6, IL-1β in serum by inhibiting the activation of astrocytes and microglia, reduce the generation of Aβ and amyloid plaques and increase the Ach content in the cortex, thereby protecting neurons and preventing AD.	Wang et al. (2021a)
*General Records of Holy Universal Relief*	Yuanzhi Powder		Reinforce functional activities of the heart, Treating forgetfulness Inhibiting of AchE activity in the brain, Increasing the activity of antioxidant enzyme system in the brain, Anti-oxidative stress damage.	Li et al. (2017), Guo et al. (2019)
*Valuable Prescriptions for Emergency*	Buwang Powder, Jiaweibuwang Powder	α-Asarone, β-asarone, Eugenol	Modulating Aβ aggregation and reducing its induced neurotoxicity, Regulating the acetylcholinergic system, Inhibiting neuroinflammation, oxidative stress and hippocampal neuronal apoptosis, and promoting neuronal regeneration, maintaining synaptic plasticity, reducing glutamate excitotoxicity and intracellular calcium overload to improve learning memory impairment.	He et al. (2020)
Empirical formulas	Bingchang Powder	Volatile oil mixtures of *Acorus tatarinowii*, *Eugenia caryophyllata* Thunb., *Ligusticum chuanxiong hort*	Treating forgetfulness Significantly reducing Aβ plaques in the brain tissue of model mice, reducing the concentration of TNF-α and inhibiting the expression of caspase-3 in the hippocampal CAI region to ensure the number and structural integrity of neuronal cells. Activating the SIRT1/NF-κB signaling pathway.	Zhou (2021)
Persian Traditional Medicine	*Pimpinella anisum L.* Apiaceae	anise oil	Neuroprotective effect	Karimzadeh et al. (2012)
India	*Nardostachys jatamansi* DC.	Volatile oils	Antioxidant, anti-neuroinflammatory effects, neuroprotective effects in CI.	Rao et al. (2012), Panara et al. (2020)
Iran	*Tetraclinis articulata*	Volatile oils	Restoring oxidative-antioxidant status and inhibiting AchE activity	Sadiki et al. (2019)
All around the world	*Lavandula angustifolia* Mill.	Volatile oils	Relieving dementia agitation	

## 4. Mechanism of volatile oil for cognitive impairment

**Figure 1:**
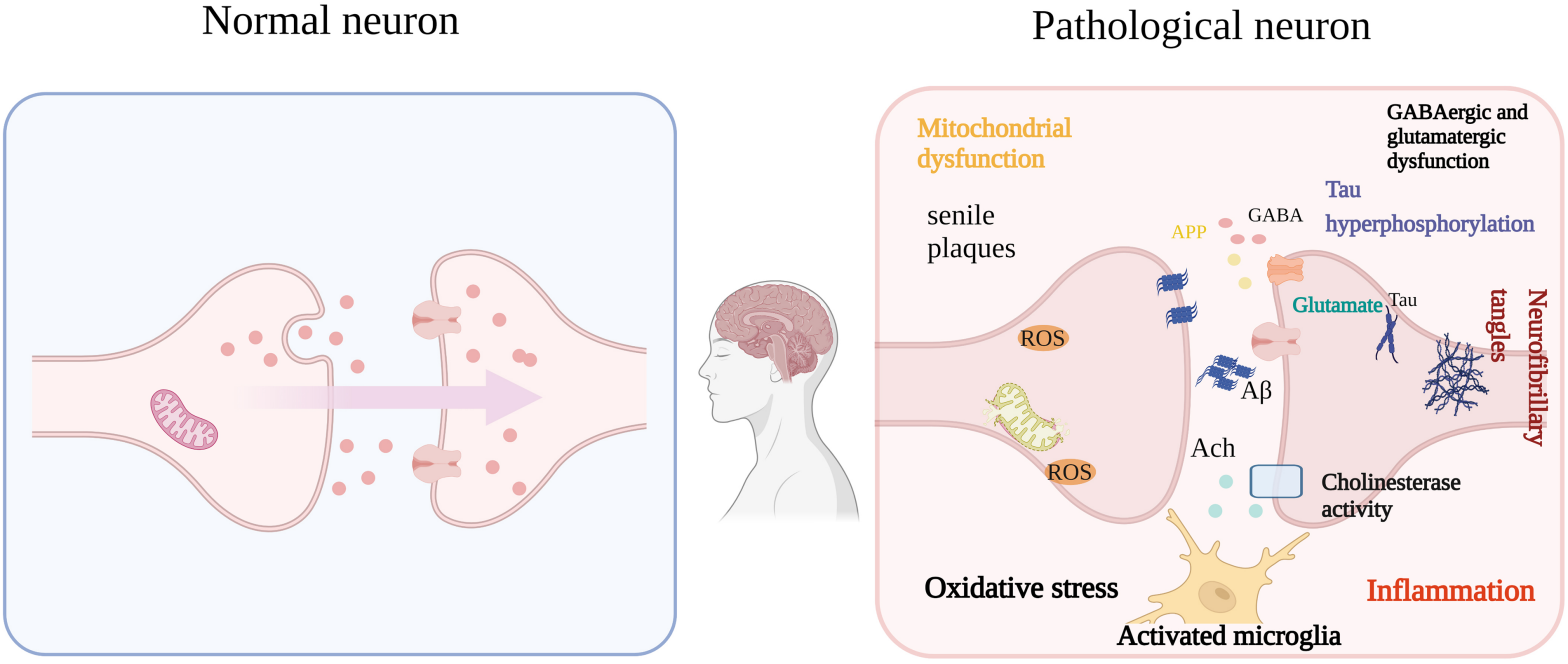
Aβ, NFTs, OS, etc. are the main causes of IC (e.g.: AD). The self-aggregation of Aβ and NFTs formed by abnormal aggregation of tau protein in the brain, functional degradation of central cholinergic neurons, and their resulting OS, microglial activation and proinflammatory cytokine release. The vicious circle eventually leads to cognitive impairment.

**Figure 2:**
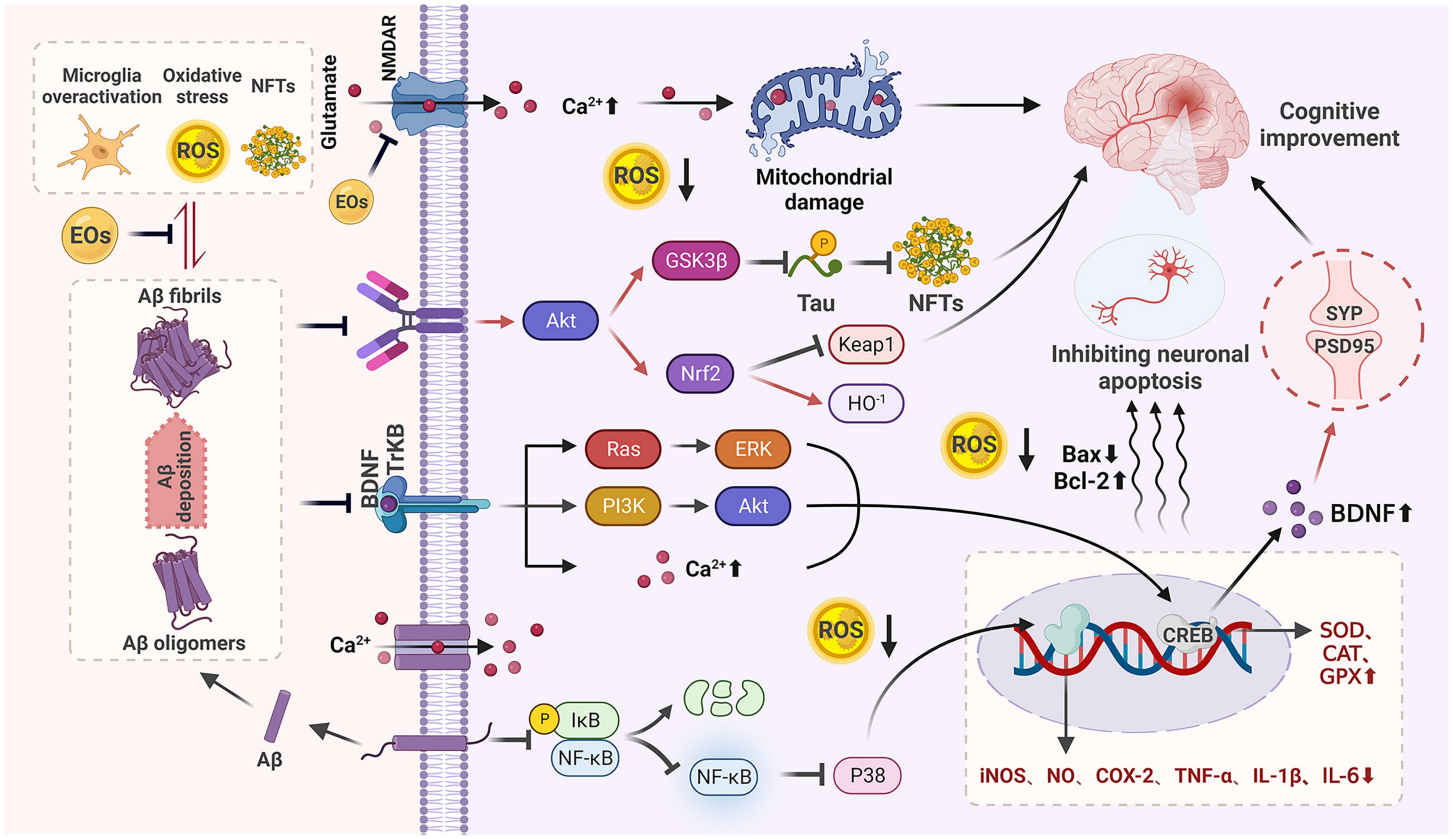
Anti-CI mechanism of natural EOs. Natural EOs play a role by reducing Aβ neurotoxicity, anti-oxidative stress, improving microglia-mediated neuroinflammation, regulating BDNF, and inhibiting neuronal apoptosis.

**Figure 3:**
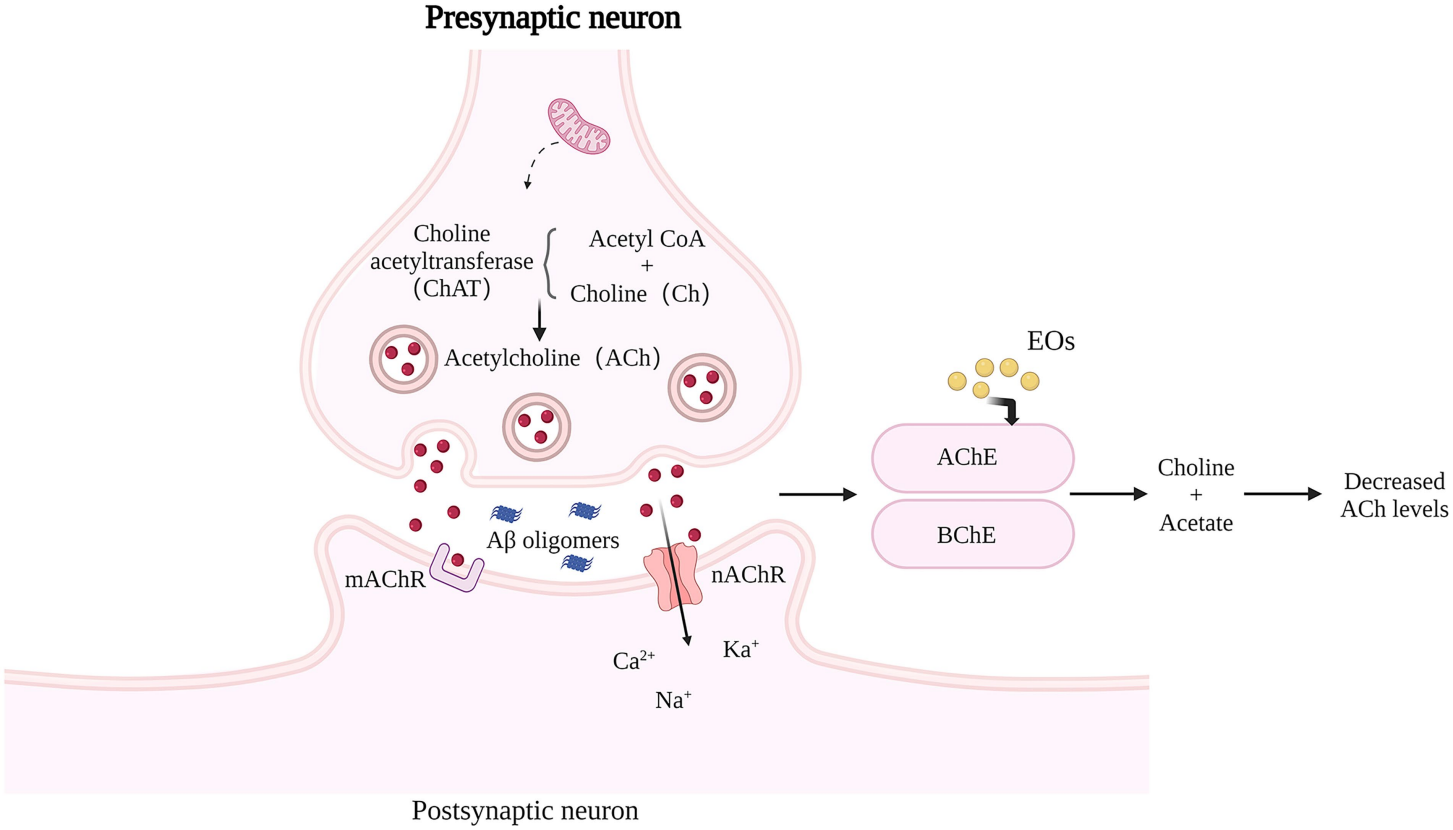
Effects of Natural EOs on brain cholinesterase activity. AChE or BChE decomposes ACh into choline and acetate. ACh level in AD brain is low, and cholinergic nerve transmission is impaired. Natural EOs significantly enhanced the specific activity of AChE, and increased the amount of ACh that remained in the synaptic space and interacted with postsynaptic receptors.

SP caused by Aβ self-aggregation, NFTs formed by abnormal tau protein aggregation and the chronic inflammation and oxidative stress they all can lead to neuronal degeneration, neuronal apoptosis, and CI. In addition, severe deletion, degeneration and functional defects of cholinergic neuronal sites, which damage acetylcholinergic nerves, also lead to learning memory impairment and CI. It can be seen that neurotoxicity of Aβ, NFTs, cholinergic system dysfunction, oxidative stress, and chronic inflammation are the main causes of cognitive dysfunction (Figure 1), and natural plant volatile oil treatment improves CI mainly through these pathways (Figures 2, 3).

### 4.1. Mitigation of beta amyloid peptide neurotoxicity

Aβ is a 36–43 amino acid polypeptide produced by β-secretase (also known as β-site amyloid cleavage enzyme, BACE) and γ-secretase mediated cleavage of amyloid precursor protein (APP; Chen and Yan, 2010), The most abundant forms of Aβ are the residue 40 and 42 peptide variants Aβ1-40 and Aβ1-42, with Aβ 42 being more hydrophobic at the C-terminus and more likely to accumulate in brain tissue and cause disease. Soluble Aβ aggregates are usually classified as oligomers and protofibrillar proteins (Tiwari et al., 2019). The main component of amyloid plaques in the brain is the larger amyloid fibrils aggregated by Aβ monomers. It has been shown that Aβ monomers do not directly affect neuronal function, but the soluble oligomers produced after monomer hydrolysis are the key factors affecting cognitive function in AD patients (Tu et al., 2014). For example, Aβ oligomers can bind to N-methyl-D-aspartic acid receptor (NMDAR) to increase Ca^2+^ concentration in neuronal cells, which leads to increased intracellular oxidative stress and dendritic spine loss, resulting in neuronal cell death (Birnbaum et al., 2015; Huang et al., 2015) and it has also been shown that Aβ-formed amyloid fibrils can enter the hydrophobic layer of the cell membrane and cause cell membrane damage by inducing cytoskeletal protein cross-linking (Sasahara et al., 2014). The accumulation of fibrillar amyloid in the brain of AD patients can cause permanent destruction of synapses, leading to cognitive and memory loss (Forny-Germano et al., 2014).

In addition, there is growing evidence that the imbalance of Aβ production and clearance in the brain of AD patients is a central issue contributing to the development of AD. Under physiological conditions, Aβ produced by neurons has multiple degradation pathways, including clearance by glial cells, degradation by proteases, transport by LRP-11 mediated by vascular endothelial cells, or eliminated *via* perivascular drainage pathways. APOE is a plasma protein involved in cholesterol transport, synthesized mainly by the brain and liver, involved in the regulation of Aβ production and influencing the clearance of Aβ by neurons and astrocytes (Liu and Cao, 2020). When aging and multiple risk factors reduce or disrupt the body’s ability to clear Aβ, resulting in the untimely deposition of Aβ produced by neurons in different parts of the brain, the abnormal accumulation of Aβ in the brain can damage the structure and function of neuronal cells, participate in oxidative damage, accelerate cellular aging, and ultimately lead to cognitive decline (Cheng et al., 2020; Wang et al., 2020). Aβ can also promote other pathophysiologies, such as increasing tau protein phosphorylation and thus promote neurogenic fiber tangling process, induce apoptosis of neuronal cells, cause cholinergic neurological damage, induce oxidative stress to increase reactive oxygen species (ROS) production, damage biomolecules, promote mitochondrial energy disorders also stimulate microglia and astrocytes to release large amounts of pro-inflammatory cytokines, turning an acute response under normal conditions into chronic inflammatory damage, etc.

lemon EO of citrus origin (Liu et al., 2020) was able to inhibit the accumulation of amyloid and reduce neuronal loss, while improving learning and memory after neurodegeneration in APP/PS1 mice. REO (Zhu et al., 2017) significantly suppressed Aβ deposits and reduced the Aβ oligomers to alleviate the toxicity induced by Aβ overexpression, Further, REO markedly activated the expression of GST-4 gene, which supported that REO reduced Aβ oligomers to treat AD worms through SKN-1 signaling pathway. It has been shown that a certain concentration of *Acorus tatarinowii* Schott EO (Ma et al., 2007) can effectively convert Aβ 25–35 from α-helix to β-fold, affecting its secondary structure and thus preventing Aβ aggregation and fibril formation. Intervention of APP/PS1 mice with the active ingredient of Acorus tatarinowii Schott, β-Asarone, revealed that the expression of p-mTOR and p62 was reduced in the treated group and the expression of p-Akt, Beclin-1, and LC3B was reduced in the treated group compared to the blank group, indicating that β-Asarone could inhibit Beclin-1 and LC3B by upregulating the PI3K/Akt/mTOR signaling pathway to inhibit Beclin-1-dependent autophagy to attenuate Aβ1-42-induced neuronal toxicity and improve cognitive performance in AD mice (Xue et al., 2014; Deng et al., 2016). β-Asarone can also reduce Aβ production by inhibiting the level of APP expression in the hippocampus and cortical layer of the brain in demented mice, exerting a protective and restorative effect on learning-related synapses in the hippocampus (Zhang et al., 2014). In addition, Oxyphylla A (from *Alpinia oxyphylla* Miq. plant EO; Bian et al., 2021) was able to reduce APP and Aβ protein expression levels and improve cognition by exerting antioxidant effects through the Akt-GSK3β and Nrf2-Keap1-HO-1 pathways, and Limonene (Piccialli et al., 2021) counteracts the increase in ROS production triggered by Aβ1-42 oligomers, thus preventing the upregulation of KV3.4 activity and cell death in primary cortical neurons, exerting a neuroprotective effect. Linalool (from *Lavandula angustifolia* EO, *Melissa officinalis* EO, *Rosmarinus officinalis* EO, *Cymbopogon citratus* EO; Sabogal-Guáqueta et al., 2016) delayed cerebral amyloidosis, including amyloid deposits and β-amyloid peptide abundance, reduces intracellular ROS production, significantly decreases the levels of IL-1β, iNOS, COX-2, p38 MAPK, and exerts anti-inflammatory and antioxidant beneficial effects on AD. These EOs and their components exert neuroprotective effects against Aβ-induced toxicity through anti-amyloid, anti-inflammatory and antioxidant effects.

### 4.2. Mitigation of oxidative stress damage

Regarding the pathogenesis of CI, many studies have confirmed the association with oxidative stress damage. Oxidative stress is not only involved in the initial stages of AD, but also influences disease progression by activating various cellular signaling pathways that lead to the formation of toxic substances (Veurink et al., 2020). OS response refers to a state of imbalance between oxidation and antioxidant in the body that can lead to the accumulation of ROS, and with a large accumulation of ROS, oxidative damage reactions can occur to macromolecules, which in turn can cause damage or loss of tissue and organ function. The brain usually requires higher levels of oxygen to perform its extensive synaptic functions and is highly susceptible to oxidative stress, especially in hippocampal and cortical regions (Kamat et al., 2016). Under normal physiological conditions, antioxidant enzymes are able to overcome oxidative stress generated *in vivo* (Beckhauser et al., 2016), but in AD patients and aging brains, the accumulation of Aβ, mitochondrial dysfunction, etc. leads to elevated ROS levels, which further promote the aggregation of Aβ due to oxidative damage severely affecting the function of various proteins, enzymes, lipids and ion channels thus causing neurotoxicity decreasing hippocampal plasticity and directly participating in the pathogenesis of AD (Wang et al., 2023). In addition, oxidative stress can release a variety of cytotoxic substances that activate microglia and directly trigger neuronal damage and death (Knezevic and Mizrahi, 2018), and with the activation of apoptosis and the reduction of antioxidant enzymes, the accumulation of ROS/RNS has a catastrophic effect on cholinergic areas involved in cognitive performance, ultimately leading to the development of cognitive dysfunction. There are many endogenous antioxidant enzymes, HO-1 being the most potent one, and Nrf2, an upstream transcription factor that regulates HO-1 expression, and antioxidant therapies based on Nrf2 and HO-1 targets may be useful in CI prevention and treatment (Ali et al., 2018; Osama et al., 2020). Free radicals generated during oxidation are neutralized to non-free radical forms by antioxidant enzymes such as CAT, SOD, GPX. However, when free radical production is abnormally high or the immune system is depleted, free radical scavengers need to be given externally. Many plant EOs have good antioxidant, free radical scavenging effects. *Chimonanthus nitens* Oliv. EO (Wang et al., 2021c), *Schisandra chinensis* Baill. EO (Yang et al., 2018), *Tetraclinis articulata* EO (Sadiki et al., 2019), *Pinus halepensis* EO (Postu et al., 2019) and other EOs of various plants, etc. all showed strong antioxidant effects in CI, increased the activity levels of SOD, CAT and GPX in hippocampal tissues, and significantly improved the Aβ1-42-induced decrease in GSH levels, increase in protein carbonyl and MDA, thus reducing oxidative stress and improving Aβ1-42-induced memory impairment in rat hippocampus. In addition, the terpenoids such as α-Cyperone (Huang et al., 2018), 1,8-cineole (Khan et al., 2014), Linalool (Yuan et al., 2021), Thymoquinone (Abulfadl et al., 2018), Terpinolen (Bahareh et al., 2020), oxyphylla A (Bian et al., 2021) etc. in EOs were able to increase antioxidant enzyme activity *in vivo* and improve cognition by ameliorating oxidative stress. Studies have shown that α-Cyperone and oxyphylla A antioxidant mechanisms are associated with increased expression of Nrf2 and its downstream genes HO-1 and NQO1 in the brain and inhibition of Nrf2 regulatory protein Keap1 expression, which exert antioxidant effects through the Akt/Nrf2-Keap1-HO-1 pathway to improve cognition.

### 4.3. Regulation of the central cholinergic system

The central cholinergic nervous system (CNS), plays an important regulatory role in cognitive functions such as learning and memory, and some studies have shown that reduced neuronal activity due to degeneration of cholinergic neurons is one of the pathological factors for the appearance of symptoms in patients with cognitive dysfunction (Hampel et al., 2018). Intracerebral acetylcholine (ACh) is present in the vesicles of cholinergic neurons and is the neurotransmitter most closely related to learning and memory identified so far (Mesulam, 2013), it conducts signals related to cognition, learning and memory, and its metabolic processes are closely related to AD. A decrease in the number of central cholinergic neurons, a decrease in ACh synthesis, and a decrease in ACh receptors may lead to learning memory impairment. ACh is synthesized from choline and acetyl coenzyme A catalyzed by Recombinant Choline Acetyltransferase (Hampel et al.) and is rapidly hydrolyzed by acetylcholinesterase (Doody et al., 2007) once released from the vesicles. ChAT and AchE work together to maintain the dynamic balance of ACh. Clinical studies have found that the degree of CI in VD patients is associated with a decrease in ACh synthesis and a relative increase in AChE activity, and in particular, a sustained decrease in hippocampal ACh content may be an important factor in the development of VD (Watanabe et al., 2008). The increased central AchE content and the disruption of the body’s antioxidant enzyme system severely affect the cognitive pathways in MCI patients. In the neocortex and hippocampus, ACh is not only involved in the activity of a large number of neurons, but also regulates synaptic plasticity.

Partial ChEI increase the availability of acetylcholine at brain synapses and are one of the few pharmacological therapies clinically proven to treat CI. Capatina et al. (2020) studied a zebrafish (Danio rerio) model of memory impairment induced by scopolamine (Sco) and found a significant increase in AChE-specific activity in sco-treated zebrafish compared to controls, While TEO treatment resulted in a significant decrease in AChE-specific activity. Aazza et al. (2011) demonstrated that TEO exhibited anti-AChE activity mainly due to the presence of the phenolic monoterpenes thymol and carvacrol. In addition, EOCO (Bae et al., 2012), AOEO (Ma et al., 2018), *Zataria multiflora* Boiss. EO (Majlessi et al., 2012), all significantly inhibited AchE activity and increased Ach levels in the brain and reduced neuronal apoptosis. *Rosmarinus officinalis* EO (EORO) produced a significant improvement in the rate of spontaneous alternation behavior, improving cognitive function by activating the central nervous system. Bergapten (Kowalczyk et al., 2020), β-asarone (Saki et al., 2020), Thymoquinone (Abulfadl et al., 2018) and other active components of essential oils were also able to inhibit AChE activity in the brain. Boiangiu et al. (2020) made a blend of essential oils (MO) with limonene (91.11%) as the main chemical component and studied its cognitive facilitation effect on scopolamine-induced amnesia in rats. It was shown that MO inhibited the oxidative stress state and the activity of AChE and BChE in the brain of model mice and improved the memory impairment induced by Sco by restoring the activity of the cholinergic system and the antioxidant status of the brain.

### 4.4. Reducing inflammation

Numerous studies have shown that inflammation has an important role in the pathogenesis of AD, inflammatory processes may promote neuronal loss and cognitive decline (Hayes et al., 2004; Cunningham et al., 2005; Kim and Joh, 2006), brain inflammation appears to play a neuroprotective role in the acute phase response but becomes detrimental in the chronic response to toxic injury (Kim and Joh, 2006). Inflammatory inducers, such as LPS, activate microglia to promote the degradation of Aβ. Impaired microglia function promotes the progression of AD and leads to increased Aβ accumulation in the brain (Choi et al., 2009; Xiao et al., 2011). In addition to providing beneficial effects to the host, Aβ or APP-activated microglia release a variety of pro-inflammatory and toxic products, including ROS, nitric oxide (NO), and cytokines such as interleukin 1β (IL-1β), interleukin 6 (IL-6), and tumor necrosis factor α (TNF-α), ultimately leading to an increased inflammatory response and severe neuronal loss (Sajja et al., 2016). In turn, elevated IL-1β levels exacerbate the accumulation of Aβ (Goldgaber et al., 1989; Heneka et al., 2015) and increase the production of other cytokines (e.g., IL-6) thereby activating CDK-5 kinase and leading to tau hyperphosphorylation (Quintanilla et al., 2004). This means that microglia have the same dual function in the protection of cognition. Some evidence suggests that long-term use of NSAIDs reduces the risk of AD and delays disease progression, possibly through the inhibition of cyclooxygenase (COX) and activation of peroxisome proliferator-activated receptor gamma (PPARγ; Gasparini et al., 2004), thereby decreasing prostaglandin synthesis and reducing cytokine secretion.

Mohamed et al. (2021) investigated the AD-related anti-inflammatory activity of SO, and found that SO significantly improved AlCl3-induced learning and memory impairment in mice, decreased AChE and Aβ levels, down-regulated TNF-α and IL-1β, decreased NF-κB and p38MAPK expression levels, and increased BDNF and PPAR-γ expression, It was shown that SO attenuated neuroinflammation and promoted cognitive recovery by regulating NF-κB/p38MAPK/BDNF/PPAR-γ signaling pathway. Ligusticum chuanxiong hort EO (Zhou et al., 2019), reduced the level of inflammatory factors TNF-α and NO, and its mechanism of action to improve CI in VCI mice may be related to inhibition of brain inflammatory response and reduction of neuronal damage. Phenylallyl compounds in Cinnamomum cassia Presl significantly inhibited the increase in COX activity and prostaglandin E2 release caused by IL-1 stimulation of brain microvascular endothelial cells, which in turn improved the memory function of APP transgenic AD mice (Ran et al., 2017). In addition, *Schisandra chinensis* (Turcz.) Baill. EO (Xu et al., 2019) was able to reduce the phosphorylation of p-38, attenuate the release of pro-inflammatory cytokines IL-1β, 1L-6, and TNF-α, and ameliorate microglia hyperactivation by inhibiting the activation of NF-κB/MAPK pathway. 1,8-cineole (Khan et al., 2014), Linalool (Yuan et al., 2021), and β-caryophyllene (Sudeep et al., 2021) in the essential oils of the plant were able to reduce the levels of the pro-inflammatory markers IL-1β, iNOS, COX-2, and p38 MAPK. α-Cyperone (Huang et al., 2018) from Cyperus rotundus L. EO was able to upregulate Nrf2, HO-1, p-Akt, and downregulate p-NF-κB, p65, TNF-α, IL-6, IL-1β. It inhibited inflammatory cytokine production in BV-2 cells by activating Akt/Nrf2/HO-1 and inhibiting NF-κB pathway and thus exerted neuroprotective effects.

### 4.5. Other mechanisms

Hyperphosphorylated Tau-rich neurofibrillary tangles (NFTs) are another neuropathological hallmark of AD. Under physiological conditions, Tau is the most abundant microtubule-related protein in neurons, mainly concentrated in neurons in the frontal, temporal, hippocampal and entorhinal regions of the brain, as well as in the axons of peripheral nerves (Gao and Kong, 2016). Its role in initiation and stabilization in the assembly of microtubules. This is critical for axonal transport and neuronal function (Wang and Tian, 2012). While the hyperphosphorylation of Tau protein accelerates its accumulation in the brain and cerebrospinal fluid and directly promotes the formation of NFTs, the hyperphosphorylated Tau protein competes with microtubule proteins such as MAP1 and MAP2 to bind microtubules leading to microtubule depolymerization and hindering axoplasmic transport, resulting in reduced binding of Tau protein to microtubule proteins, and the Tau proteins shed from microtubule proteins aggregate with each other to form the fibrillar material with neurotoxic properties, NFTs (Horvath et al., 2014). NFTs can reduce cis-axonal transport without altering microtubule integrity, which in turn induces neuronal degeneration and ultimately leads to cognitive decline and dementia. Once NFTs are formed, they can spread to other areas of the brain. Moreover, the abnormal secretion and accumulation of Aβ in neuronal cells can overactivate Tau protein kinase and promote Tau protein phosphorylation, which in turn triggers a chronic inflammatory response, activates apoptosis, generates incomplete metabolized free radicals, and causes an imbalance between intracellular oxidative and antioxidant effects in neurons, resulting in the death of a large number of neurons and glial cells (Nussbaum et al., 2012). Thus, a therapeutic approach to the clearance of Tau aggregates appears to be a viable way to reduce their pathology (Selvarasu et al., 2022; Sreenivasmurthy et al., 2022), and one study found that Tetrandrine, a medicinal natural product derived from *Stephania tetrandra* S. Moore, enhances autophagy-lysosomal pathway (Panara et al., 2020) function and reduces pathological Tau by Enhancement of glial cell clearance and reduction of pathological Tau transmission (Tong et al., 2022). It has been found that various plant EOs can act as a strategy to improve CI by preventing Aβ deposition and Tau hyperphosphorylation. Jeon et al. (2011) found that early inhalation of SHXW EO improved CI caused by Aβ (1–42) and inhibited Aβ-induced Tau phosphorylation by suppressing the activation of JNK and p38 in the brain. Lee et al. (2021) fed AD mice Induced by Aβ1-40 with the same essential oil content of *Litsea cubeba* Persoon Powder, and showed that it reduced P-Tau content and Aβ plaques, resulting in about 3–8% reduction in brain atrophy.

Several studies have shown that Aβ downregulates response element binding protein (CREB)-mediated transcription (España et al., 2010; Pugazhenthi et al., 2011), that CREB-mediated gene expression is impaired in AD brains, and that CREB-regulated BDNF levels are reduced.BDNF is the most widely distributed neurotrophic growth factor in the CNS and plays an important role in brain regions involved in learning, memory, and higher cognitive functions (Bekinschtein et al., 2008). activation of CREB promotes transcription of key proteins of activity-dependent plasticity, particularly BDNF. Decreased levels of BDNF may contribute to the degeneration of specific neuronal populations and the progressive atrophy of neurons in AD-affected brains (Cowansage et al., 2010). Altered BDNF expression levels or disruption of the BDNF–TrkB signaling pathway may lead to synaptic loss and cognitive dysfunction (Song et al., 2015). Studies have shown that even after clinical onset of AD, increasing the expression of BDNF by modulating the mRNA level of CREB may spare patients from memory deficits and cognitive dysfunction (Rosa and Fahnestock, 2015). Increasing the mRNA expression of BDNF and its receptor tyrosine kinase-coupled receptor (TrkB) in the hippocampus also improves cognition to some extent (Amidfar et al., 2018). Ma et al. studied the improvement effect of Oil extract from *Alpinia Oxyphylla* Miq.fruit (Ma, 2019; AOFOE) on scopolamine-induced learning and memory impairment in mice. The results showed that AOFOE could significantly increase the mRNA levels of BDNF and CREB in the hippocampus of learning and memory impaired mice. By regulating BDNF, CREB is further activated, thereby exerting neuroprotective effects. Lemon EO of citrus origin (Liu et al., 2020) is able to enhance memory by enhancing synaptic plasticity by increasing BDNF, PSD95 and synaptophysin. In addition, the sesquiterpene β-caryophyllene decreased the proBDNF/mBDNF ratio and was able to increase the expression of TrkB. Kim et al. (2018) found that 6-Gingerol can increase the protein expression of BDNF by activating the Akt-CREB signaling pathway.

In addition, Neuronal apoptosis is one of the important mechanisms by which many neurodegenerative diseases arise. Among the major genes regulating neuronal apoptosis, the interaction between the pro-apoptotic Bax protein and the anti-apoptotic Bcl-2 protein maintains a dynamic balance between neuronal proliferation and apoptosis. Angelica sinensis (Olive.) Diels EO (Cui, 2015), Lavandula angustifolia Mill. EO (Xu et al., 2016), AOFOE (Ma, 2019), SHXW EO (Jeon et al., 2011) and other plant essential oils can promote the expression of Bcl-2 protein with neuroprotective effect in brain tissue, reduce the expression of apoptosis Bax protein, thereby inhibiting nerve cells Apoptosis, accelerates the recovery of neural function, and together play a role in brain protection and improve cognition. Changes in central amino acid levels, especially the imbalance of Glu and γ-aminobutyric acid (GABA) levels, are key factors contributing to neuronal damage. The balance of glutamate Glu and GABA levels is important for maintaining cognitive function in the hippocampus (Sartorius et al., 2007). Studies have shown that eucalyptus oil increases brain GABA levels (Yadav et al., 2019). α-Asarone (Li et al., 2019) significantly improves behavioral performance in an ethanol-induced memory impairment model in mice by controlling calcium overload, decreasing synaptophysin I (SYN I) activity, reducing Glu release, and normalizing Glu transport function by decreasing Glu concentration and The underlying mechanism is to regulate the calcium signaling cascade to correct the function of related proteins by reducing Glu concentration and regulating the level of phosphorylated calcium/calmodulin-dependent protein kinase II (pCaMKII) to reduce the overactivity of Glu receptors AMPA and NMDA to maintain the Glu and GABA levels.

## 5. Advantages of volatile oil in the treatment of cognitive disorders

CI has many pathogenic factors, a long course, and complex pathological mechanisms that are not fully understood. Currently, the treatment for improving cognitive function in patients with MCI, VCI, diabetes-related CI, depression-related CI, and AD is based on symptomatic treatment to deal with associated risk factors and antidementia therapy. As of today, there are no medications available to slow the progression of AD (Grossberg et al., 2019), commercially available ChEI and NMDA (memantine) only provide symptomatic relief, their clinical effectiveness remains controversial, and the results of meta-analyses of their effects on behavioral outcomes are inconsistent (Doody et al., 2007; Hansen et al., 2008; Tan et al., 2014; Matsunaga et al., 2015). And ChEI often lead to adverse effects of gastrointestinal disturbances, significantly increasing the risk of dizziness, nausea, anorexia, vomiting and diarrhea, memantine is usually well tolerated but also causes adverse effects such as constipation, dizziness, headache, hypertension and drowsiness. Thus, the search and discovery of drugs and methods to improve CI remains urgent at the present time.

As a multi-causal and heterogeneous disease, it is difficult to achieve the desired effect with single drug and single target treatment for dementia, and synergistic treatment with multiple links and targets may be the future trend in drug treatment and research and development. Chinese medicine is expected to be a new source of drugs as it contains multiple active ingredients and can act on multiple targets at the same time, which is in line with the multi-factorial and multi-pathological pathogenesis of the disease. In addition, Chinese medicines have relatively low toxic side effects and are safe, and can be used in combination with Western medicines to achieve good therapeutic results. Chinese volatile oils are the most representative active components of aromatic Chinese medicine, with the main active ingredients being aldehydes and esters, monoterpenes and sesquiterpenes, and other substances that have been used for generations to alleviate the symptoms of AD and other dementias. These small molecule active ingredients are highly lipid soluble and can cross cell membranes and the BBB (Agatonovic-Kustrin et al., 2019), compensating for the low permeability and pharmacokinetic problems of some drugs. The preparation of essential oils extracted from natural plants into appropriate dosage forms and their entry into the body to exert therapeutic effects through inhalation, massage, intestinal and oral routes is known as aromatherapy. Aromatherapy is a branch of Western complementary and alternative medicine that is highly effective in improving cognition and is convenient and inexpensive, with few side effects. Aromatherapy has been tested in animals, cellular models, and clinical trials in subjects with CI to explore the underlying pathological mechanisms that reduce symptoms or affect the disease in patients with dementia (Forrester et al., 2014), and the results showed that a variety of plant essential oils showed cognitive improvement anti-dementia-related activities *in vitro* and *in vivo*, through anti-amyloid, anti-acetylcholinesterase, antioxidant, anti-inflammatory, anti-apoptotic, modulation of cell plasticity and exert neuroprotective and memory enhancing effects.

In addition, aromatic inhalation and olfaction therapy can exert its unique therapeutic advantages in improving cognition, reducing dementia agonistic behavior, and promoting memory. It has been found that olfactory function is closely related to memory, and olfactory impairment may be used as an early diagnosis of neurodegenerative diseases (Doty, 2009). The olfactory receptors on the olfactory epithelium of the nasal mucosa, after sensing aromatic molecules, produce olfactory signals that project through the olfactory bulb to the primary olfactory cortex, and then from the fibers on the primary olfactory cortical centers to the neocortex, hypothalamus, hippocampus and other olfactory secondary cortical centers to regulate central and somatic functions. Whereas the hippocampus is closely related to learning memory, olfactory impulses afferent to the hippocampus can generate olfactory memory, trigger the remodeling of neurosynapses, and directly participate in the process of organizing, recognizing, encoding, and storing learning memory as a way to improve memory and cognition. Moreover, the olfactory pathway is not affected by peripheral metabolism and BBB, and the drug is delivered to the brain non-invasively through the olfactory nerve and trigeminal nerve bypassing the BBB and entering the central nervous system directly after inhalation through the nasal cavity (Crowe et al., 2018). In a clinical study, Jimbo et al. (2009) used aromatherapy to treat 28 patients with dementia with rose-lemon EO in the morning and lavender-Orange peel EO in the evening and found that the essential oil group had a better sense of cognitively relevant spatial orientation compared to the control group. Wang (2014) observed the intervention effect of rosemary inhalation and sniffing method in patients with CI after cerebral infarction, and the results showed that conventional rehabilitation therapy combined with rosemary inhalation and sniffing had the immediate efficacy of improving cognitive function and activities of daily living in patients with CI after cerebral infarction and improving the effectiveness of rehabilitation therapy.

## 6. Conclusions and outlook

In summary, the natural drug volatile oil may play a role in improving cognition by reducing the neurotoxicity of Aβ, anti-oxidative stress, regulating the central cholinergic system, anti-acetylcholinesterase activity, improving microglia-mediated neuroinflammation, reducing inflammatory factor levels, inhibiting neuronal apoptosis, and regulating the balance of central amino acid levels. Compared to existing drugs, volatile oils from natural medicines are a unique health service resource because they are convenient and inexpensive, have fewer side effects and easily cross the blood–brain barrier. The combination of aromatic inhalation therapy, based on the olfactory pathway, can better reflect the characteristics of volatile oils, which are not affected by peripheral metabolism and BBB to deliver drugs into the brain to improve cognition, and can be used as a treatment or as an adjunctive therapy to improve CI, with clear efficacy and simple safety. However, we also realize that, at present, the research on volatile oils for AD and other cognitive disorders is still mainly preclinical studies, most of which focus on behavioral observations and related biochemical indicators, while clinical studies on aromatherapy interventions for dementia are still relatively few, and there is no unified standardized protocol for aromatherapy interventions for patients with cognitive disorders, and the intervention protocols are mostly developed by researchers themselves. Therefore, there is still a need for *in vivo*, *in vitro* experiments and clinical research to deeply explore the substance basis, action targets and mechanisms of natural plant volatile oils for the treatment of AD and other cognitive disorders, and it is believed that with the deepening of basic research and the rapid promotion of translational medicine, more effective and safe aromatic formulations will be used for the improvement and prevention of cognitive disorders. At the same time, we should also be aware that the causes of CI are diverse, with a variety of diseases such as hypertension, diabetes and depression as their risk factors. The causes and mechanisms of AD are still unclear, involving multiple signalling pathways, and there are often interconnections between different pathogenic mechanisms. Thus, the reuse or combination of existing drugs using artificial intelligence technology and network pharmacology may be a potential treatment option for AD clinical trials.

## Author contributions

NL conceived the work. YuaH reviewed the manuscript and provided corrections. AS, YL, YM, SY, DL, and JD co-wrote the paper. JW and XL prepared the figures. XH, YueH, and YW commented and corrected the paper. All authors contributed to the article and approved the submitted version.

## Funding

This work was supported by Chengdu University of Traditional Chinese Medicine Youth Foundation Advanced Talents Project (Number: QJJJ2022014), Chengdu University of Traditional Chinese Medicine Rural Revitalization Project (Number: XCZX2022007), and Sichuan Natural Science Foundation (Number: 2022NSFSC1406).

## Conflict of interest

The authors declare that the research was conducted in the absence of any commercial or financial relationships that could be construed as a potential conflict of interest.

## Publisher’s note

All claims expressed in this article are solely those of the authors and do not necessarily represent those of their affiliated organizations, or those of the publisher, the editors and the reviewers. Any product that may be evaluated in this article, or claim that may be made by its manufacturer, is not guaranteed or endorsed by the publisher.

## Figures and Tables

**Table:** 

2VO	Permanent ligation of bilateral common carotid arteries
5-HT	5-Hydroxytryptamine
5-HT3	5-Hydroxytryptamine3
AChE	Acetylcholinesterase
AChEI	Acetylcholinesterase inhibitor
AD	Alzheimer’s disease
Akt	Protein kinase B
AlCl_3_	Aluminum trichloride
AMPA	α-Amino-3-hydroxy-5-methyl-4-isoxazole-propionic acid receptor
AOFOE	Alpinia Oxyphylla Miq.fruit EO
APOE	Apolipoprotein E
APP	Amyloid precursor protein
ASD	Autism Spectrum Disorder
ATSEO	Acorus tatarinowii Schott EO
Aβ	Amyloid beta
BACE	β-Site amyloid cleavage enzyme(β-secretase)
Bax	Bcl-associated X
BBB	Blood–brain barrier
Bcl-2	B-cell lymphoma-2
BDNF	Brain-derived neurotrophic factor
CAT	Catalase
CDK-5	Cyclin-dependent kinase-5
ChAT	Recombinant Choline Acetyltransferase
ChE	Cholinesterase
ChEI	Cholinesterase inhibitor
CI	Cognitive impairment
COX	Cyclooxygenase
COX-2	Cyclooxygenase-2
CREB	cAMP-response element binding protein
DA	Dopamine
D-gal	d-GALACTOSE
EO	Essential oil
EOCO	Chamaecyparis obtusa Sieb. & Zucc. EO
EORO	Rosmarinus officinalis EO
ERK	Extracellular regulated protein kinases
FTD	Frontotemporal dementia
GABA	Gamma amino butyric acid
GFAP	Glial fibrillary acidic protein
Glu	Glutamate
GPX	Glutathione Peroxidase
GSK-3β	Glycogen synthase kinase-3β
GST-4	Glutathione S-transferase-4
HCl	Hydrochloride
HO-1	Heme oxygenase-1
i.a.	Inhalation
i.g.	Intragastric
IL-1β	Interleukin-1β
i.p.	Intraperitoneal
Icam	Intercellular adhesion molecule
iNOS	Inducible nitric oxide synthase
JNK	c-Jun Loxl2
MAO	Monoamine oxidase
Keap1	Keleh-like ECH-associated protein
LDH	Lactate dehydrogenase
LPS	Lipopolysaccharide
LRP-11	Low-density lipoprotein receptor related protein-11
MAPK	Mitogen-activated protein kinase
MCI	Mild cognitive impairment
MDA	Malondialdehyde
mTOR	Mammalian target of rapamycin
NGF	Nerve growth factor
NMDAR	N-methyl-D-aspartic acid receptor
NO	Nitric oxide
Nrf2	Erythroid-derived 2-related factor 2
OPC	Oligodendrocyte precursor cell
p38 MAPK	p38 Mitogen-activated protein kinase
PDI	Protein disulfide isomerase
PHFs	Pair helical filaments
PI3K	Phosphatidylinositol-3-kinase
PKC	Protein kinase C
polyQ	Polyglutamine
PSD-95	Postsynaptic density protein-95
REO	Rosa rugosa Thunb. EO
ROS	Reactive oxygen species
Sco	Scopolamine
SO	Sesamum indicum L. EO
TEO	Thymus vulgaris L. EO
TNF-α	Tumor necrosis factor-α
VCI	Vascular cognitive impairment
VD	Vascular dementla
VPA	Valproic acid
